# The Role of Ovarian Granulosa Cells Related-ncRNAs in Ovarian Dysfunctions: Mechanism Research and Clinical Exploration

**DOI:** 10.1007/s43032-025-01854-2

**Published:** 2025-04-02

**Authors:** Liuqing Liu, Yanyan Fang

**Affiliations:** 1 College of Traditional Chinese Medicine, Anhui University of Chinese Medicine, Hefei, 230012 China; 2 Key Laboratory of Xin’an Medicine of the Ministry of Education, Anhui University of Chinese Medicine, Hefei, 230012 China

**Keywords:** NcRNAs, MiRNAs, PiRNAs, LncRNAs, CircRNAs, Ovarian dysfunctions

## Abstract

Ovarian dysfunctions, encompassing conditions such as polycystic ovary syndrome (PCOS), premature ovarian failure (POF), premature ovarian insufficiency (POI), and diminished ovarian reserve (DOR), are closely linked to disruptions in follicular development, often tied to granulosa cell (GC) abnormalities. Despite ongoing research, the precise mechanisms underlying these dysfunctions remain elusive. Increasing evidence highlights the pivotal role of non-coding RNAs (ncRNAs) in the pathogenesis of ovarian dysfunctions. As transcripts that do not encode proteins, ncRNAs are capable of regulating gene expression at various levels. They influence GCs by modulating key biological processes including proliferation, apoptosis, autophagy, cell cycle progression, steroidogenesis, mitochondrial function, inflammatory responses, and aging. Disruptions in GC development and function can lead to impaired follicular development, consequently contributing to ovarian dysfunctions. Thus, ncRNAs are likely integral to the regulatory mechanisms underlying these pathologies, exhibiting distinct expression patterns in affected individuals. This review delves into the regulatory roles of ncRNAs in GCs and their implications for ovarian dysfunctions (PCOS, POF, POI, DOR), offering insights into potential biomarkers for ovarian function assessment and novel therapeutic approaches for treating these conditions.

## Introduction

The ovaries perform essential roles in producing and releasing oocytes, as well as synthesizing and secreting steroid hormones. Disruptions in these functions can result in various ovarian dysfunctions, including polycystic ovary syndrome (PCOS), premature ovarian failure (POF), primary ovarian insufficiency (POI), and diminished ovarian reserve (DOR). These conditions are closely linked to disorders in follicular development, which can significantly affect women’s fertility and overall reproductive health.

The follicle, the ovary’s fundamental reproductive unit, comprises one oocyte and two types of somatic cells: granulosa cells (GCs) and theca cells [1]. GC, as a critical component of the follicle, provide vital physical and hormonal support to the oocyte and play a pivotal regulatory role across various stages of follicle development, including primordial follicle quiescence, activation and entry into growth, follicle maturation, ovulation, corpus luteum formation, and atresia [2, 3]. During the initial stages of follicle development, flat pre-granulosa cells envelop the oocytes, protecting them from apoptosis [4]. As GCs transition from a flat to a cuboidal shape, the dormant primordial follicle is activated and begins to grow, at which time GCs and oocytes form the primary follicle [4, 5]. As the oocyte grows, GCs continue to proliferate, creating a stratified layer around the oocyte. Simultaneously, under the action of GCs, theca cells were gradually recruited and further differentiated into the inner and outer thecal layers, making the follicle’s progression to the secondary follicle stage. As follicular fluid accumulates, forming a cavity, the follicle advances to the antral follicle stage. At this stage, GCs differentiate into cumulus granulosa cells (CCs) surrounding the oocyte and mural granulosa cells (mGCs) forming the outer layer [1]. Under the influence of follicle-stimulating hormone (FSH), these cells produce estrogen, which further drives follicular development [6]. GCs maintain the oocytes in meiotic arrest until the pre-ovulatory phase [7], after which luteinizing hormone (LH) acts on the outer layer of mGCs [7, 8], which then receive and transmit the ovulation signal to the CCs and oocyte [9], enabling the oocyte to resume the first meiotic division and undergo ovulation [10]. Following ovulation, the CCs and oocyte migrate into the fallopian tube, while mGCs remain in the post-ovulatory follicle to form the corpus luteum, sustaining the function of ovarian luteal phase [9]. Through gap junctions, GCs supply nutrients and metabolic precursors to the oocyte [11] and produce various paracrine factors, such as growth factors, to regulate oocyte development [12]. They also signal the initiation of oocyte growth through increased protein synthesis and morphological changes. GC proliferation and differentiation are critical for oocyte growth, while GC apoptosis and deformation can lead to follicular atresia. Abnormalities in GC development and function can disrupt follicle development and cause ovarian dysfunctions [13, 14]. In conditions like PCOS [15], POF [16], POI [17], and DOR [18], aberrant GC phenotypes are often observed. Thus, investigating the regulatory mechanisms governing GC physiology and pathology is vital for enhancing the understanding of ovarian dysfunction pathophysiology and developing novel diagnostic and therapeutic strategies.

Life processes are fundamentally driven by gene regulation, and this principle applies equally to the physiological and pathological processes of GCs. The human genome comprises both protein-coding genes and numerous non-coding genes, which, while not coding for proteins, play critical regulatory roles. These non-coding RNA (ncRNA) can modulate gene expression through a variety of mechanisms at different stages, including transcription, post-transcription, and translation [19]. In recent decades, the rapid advancements in high-throughput RNA sequencing and bioinformatics have spurred a significant increase in research on ncRNAs, positioning them as a key focus for understanding disease mechanisms.

**Fig. 1:**
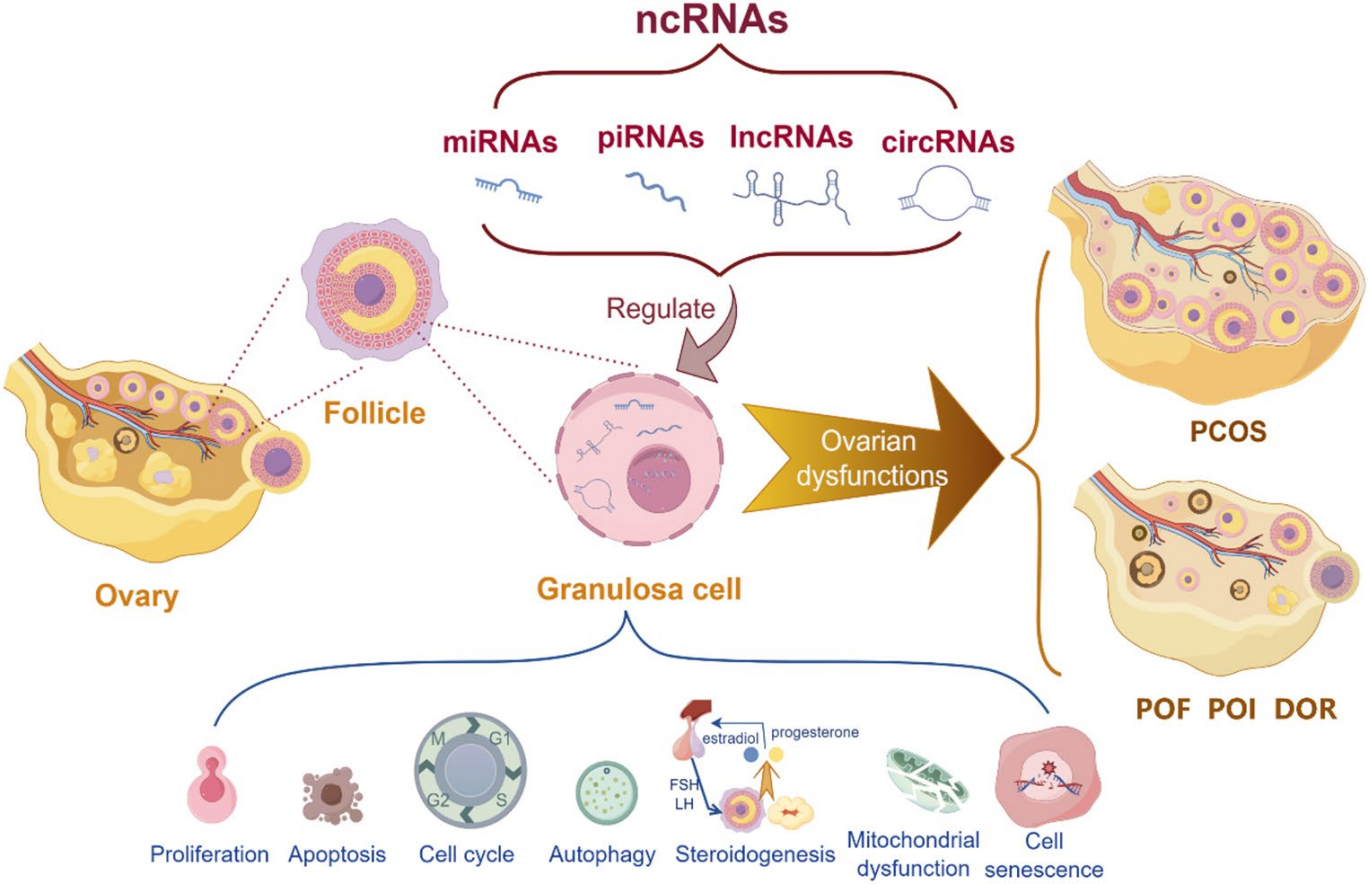
Abnormal expressions of ncRNAs can affect various GC activities and phenotypes, potentially disrupting follicular development and precipitating ovarian dysfunctions. Such dysregulation may contribute to the pathogenesis of PCOS, POF, POI, and DOR. The figure was drawn with Figdraw

The influence and regulatory functions of ncRNAs on GCs have garnered considerable attention, emerging as a crucial perspective in the study of female reproductive functions. It has been established that ncRNAs regulate various vital processes in GCs, including proliferation, apoptosis, autophagy, senescence, steroidogenesis and cell cycle progression. Given the pivotal role of GCs in these processes, the regulation of their activities and functions by ncRNAs directly impacts follicular development and is ultimately linked to various ovarian dysfunctions and female fertility issues (Fig. 1).

**Fig. 2:**
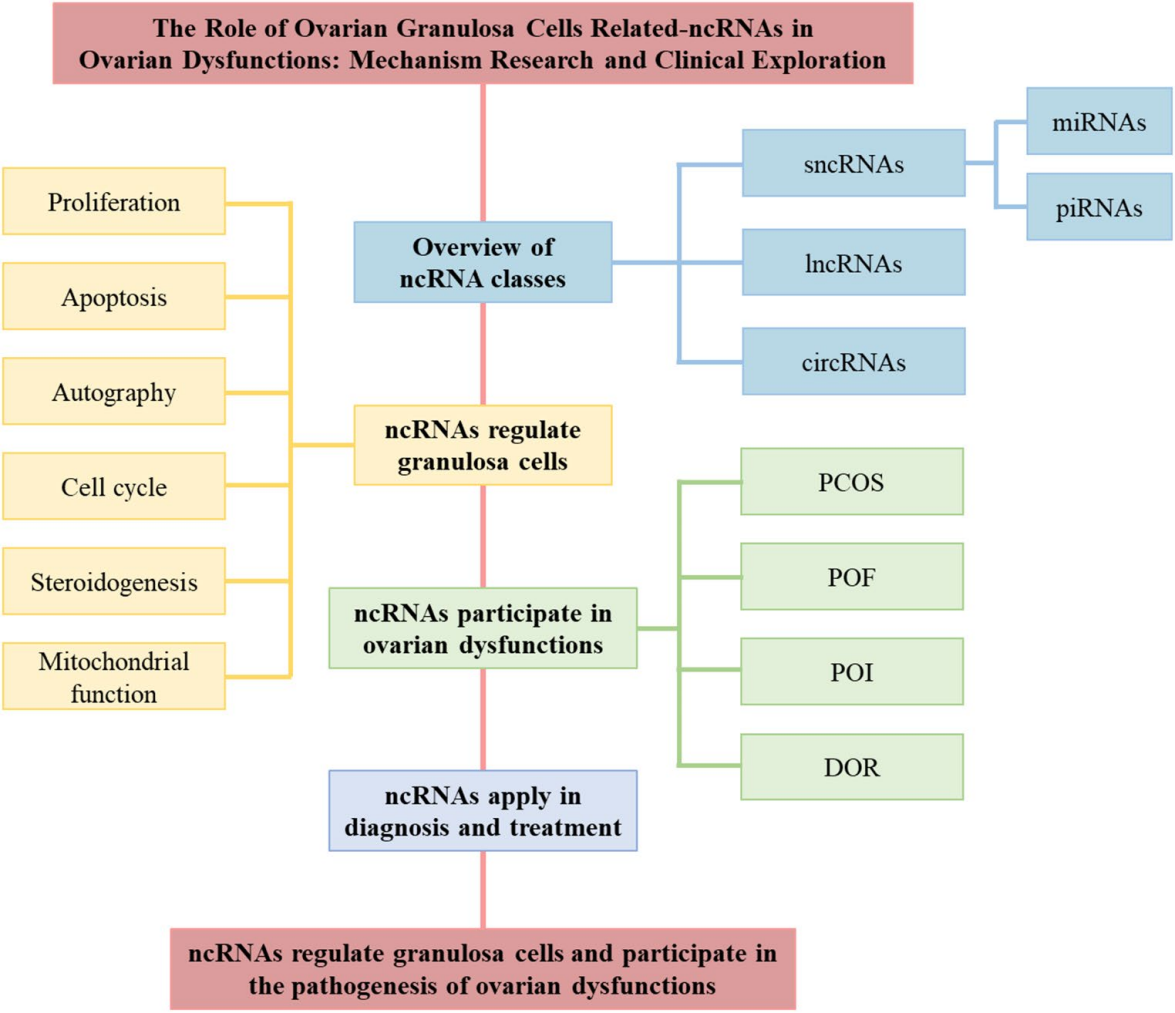
This review summarizes the regulatory effects of ncRNAs on various aspects of GCs, and elucidates the role of ncRNAs in the pathogenesis of different ovarian dysfunctions, as well as their applications in diagnosis and treatment

Extensive research has demonstrated that ncRNAs are deeply involved in the development of various ovarian dysfunctions, such as PCOS, POF, POI, and DOR. Despite the distinct symptoms and pathological features of these conditions, they all share a common thread of irregularities in follicles and GCs. While some reviews have addressed the role of ncRNAs in female reproductive endocrine dysfunctions and examined the regulatory effects of specific ncRNAs, such as microRNAs (miRNAs) and long non-coding RNAs (lncRNAs) [14, 20–23], there remains a need for a comprehensive summary of how ncRNAs influence ovarian function from the standpoint of GC dysfunction, integrating the latest research findings. This review focuses on the latest research achievements from the past three years, providing a comprehensive review of the regulatory roles of ncRNAs in various ovarian dysfunctions by influencing GC activities and phenotypes (Fig. 2). The aim of this review is to elucidate the distinct yet interconnected pathological mechanisms among various ovarian dysfunctions from the perspectives of ncRNAs and GCs. This may provide a foundation for the development of novel diagnostic and therapeutic approaches in clinical practice. Furthermore, analyzing current research methodologies and the depth of existing studies could provide a reference point and guide for future investigations.

## Search and Screening Strategies

A systematic search was conducted in PubMed (https://pubmed.ncbi.nlm.nih.gov/) using the following search strategy: ((miRNA[Title/Abstract]) OR (lncRNA[Title/Abstract]) OR (circRNA[Title/Abstract]) OR (piRNA[Title/Abstract]) OR (piwi[Title/Abstract])) AND ((cumulus[Title/Abstract]) OR (granulosa[Title/Abstract])) AND ((proliferation[Title/Abstract]) OR (apoptosis[Title/Abstract]) OR (steroidogenesis[Title/Abstract]) OR (viability[Title/Abstract]) OR (mitochondrial dysfunction[Title/Abstract]) OR (autophagy[Title/Abstract]) OR (cell cycle)). A total of 323 articles were preliminarily obtained. To obtain the latest research findings, we focused on and carefully read the literature from January 1, 2021 to June 30, 2024. And to better serve the clinical and research work related to female ovarian dysfunctions, we exclude literature unrelated to ovarian function in humans, rats, and mice. Additionally, due to the scarcity of research on piRNAs, another separate search was conducted using the search strategy: (piRNA[Title/Abstract]) AND (ovary[Title/Abstract]), without restrictions on the species of the research subjects.

## Overview of ncRNA Classes

Although ncRNAs do not encode proteins, they are capable of targeting specific genes and forming intricate regulatory networks through interactions, thereby influencing a wide range of molecular targets [24–26] and modulating various cellular processes, including chromosome dynamics, gene transcription, RNA editing, intracellular RNA transport, translation, and mRNA degradation [27–29]. Based on their length, ncRNAs are categorized into small non-coding RNAs (sncRNAs), which are less than 200 nucleotides long, and lncRNAs, which exceed 200 nucleotides. Some ncRNAs, such as circular RNAs (circRNAs), exhibit length variability and may belong to both categories [30].

### SncRNAs

#### miRNAs

Among sncRNAs, miRNAs are the most extensively studied, typically ranging from 20 to 22 nucleotides in length [20, 31]. miRNA genes are initially transcribed into primary miRNAs (pri-miRNAs), which are then processed into precursor miRNAs (pre-miRNAs) with hairpin loop structure by the activities of Drosha (a ribonuclease III-type protein). The pre-miRNAs are subsequently transported from the nucleus to the cytoplasm, where they are further processed into mature miRNAs by the modification of DICER (another ribonuclease III-type protein). Mature miRNAs are then loaded onto Argonaute (Ago) proteins to form RNA-induced silencing complexes (RISCs) [21, 32, 33], which inhibit protein expression by repressing messenger RNA (mRNA) translation or mediating degradation of target mRNAs through imperfect base-pairing with the 3’-untranslated region (3’-UTR) of the target mRNAs [31–34]. Thus, the primary function of miRNAs is to mediate gene silencing through post-transcriptional degradation and/or translational repression [35–37]. 

#### piRNAs

Piwi-interacting RNAs (piRNAs) are another class of sncRNAs, typically 24–30 nucleotides in length [38], and are associated with Piwi proteins, a subfamily of the Ago protein family [39]. Unlike miRNAs, piRNAs are produced through a Dicer-independent way [39]. When assemble with Piwi proteins, they form piRNA-induced RNA silencing complexes (piRISCs), which are transported to the nucleus [38, 40] to suppress transposon transcription [41]. The high expression of piRNAs and Piwi proteins in germline cells suggests their essential role in reproductive function [39, 42–44]. Initially thought to be exclusive to germ cells, piRNAs have also been detected in somatic cells associated with reproduction [43–45]. piRNAs and Piwi proteins play a crucial role in maintaining genomic stability and germline development by silencing specific genetic elements, which highlights their importance in the transmission of genetic information across generations [46, 47].

### lncRNAs

lncRNAs, defined as ncRNAs longer than 200 nucleotides, are notable for their heterogeneity and functional versatility. Most lncRNAs are transcribed by RNA polymerase II, and they often exhibit post-transcriptional modifications such as 5′-end m^7^G caps and 3′-end poly (A) tails [48, 49]. lncRNAs are characterized by tissue specificity, low expression tendency [50, 51], and complex, multi-dimensional evolutionary conservation [52, 53].

Current research has focused on the mechanisms by which lncRNAs regulate downstream target genes, often through the sponging of miRNAs. Additionally, lncRNAs can directly regulate mRNAs through cis- or trans-acting mechanisms, thereby affecting protein expression [54, 55]. They are also capable of modulating DNA, proteins, chromatin, and even subcellular structures [19, 48, 53, 56–58]. By influencing the activation and expression of distant [59] and neighboring genes [60], impacting RNA transcription [61] or encoding peptides [62], and participating in the regulation of subcellular structures such as mitochondria and ribosomes [48, 58, 63], lncRNAs play a role in modifying the epigenome [64].

### circRNAs

circRNAs, sometimes regarded as a subclass of long non-coding RNAs [57], originate from linear RNAs. They are formed through a progress known as back-splicing, in which the downstream 5’ splice-donor site is covalently linked to the upstream 3’ splice-acceptor site, creating a circular RNA molecule [65]. This reverse splicing mechanism, typically from protein-coding genes or other lncRNAs, results in the generation of circRNA [66–70].

Due to their covalently closed structure and the absence of 5’cap and 3’ polyadenylated [poly(A)] tails [69, 70], circRNAs exhibit high stability, rendering them resistant to ribonucleases digestion [68, 71]. circRNAs can regulate mRNA transcription and translation by sponging miRNAs and RNA binding proteins (RBPs), thereby influencing gene expression [70, 72–74]. Moreover, despite lacking the 5’ cap and 3’ poly(A) tail typically required for conventional translation initiation, some circRNAs are capable of encoding proteins with unique functions [72]. This translation is facilitated by the presence of internal ribosome entry site (IRES) elements [75, 76] or N^6^-methyladenosine (m^6^A) motifs within the circRNAs [77].

## Roles of ncRNAs in Regulating GCs

**Fig. 3:**
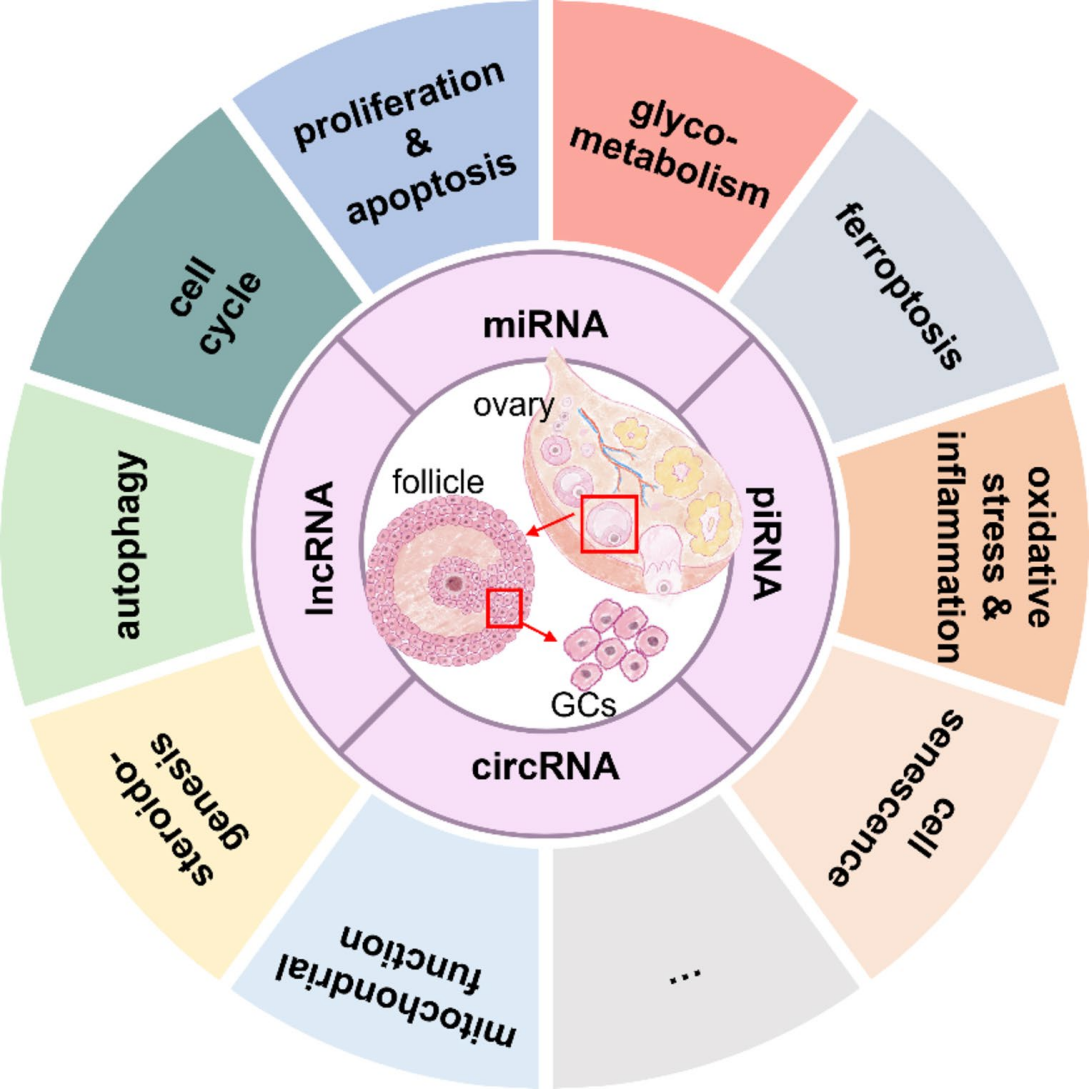
Roles of ncRNAs in regulating GCs. ncRNAs may involve in GC activities through the following mechanisms: cell proliferation and apoptosis, cell cycle progression, cell autophagy, steroidogenesis, mitochondrial function, glycometabolism, oxidative stress and inflammation, cell senescence, ferroptosis, etc. The figure was drawn with Figdraw

A variety of GC life activities, such as proliferation, apoptosis, cell cycle, autophagy, steroidogenesis, and mitochondrial function, can be regulated by ncRNAs (Fig. 3). Many studies have focused on the role of miRNAs, lncRNAs, and circRNAs, showing the complex regulation of ncRNAs on GC functions through in vitro and in vivo studies (Tables 1, 2 and 3).

**Table 1:** Regulation functions of miRNAs in GCs

Name	Sources	Related diseases	Effects on GCs	Target genes	Reference
miR-4433a-3p	GCs	PCOS	inhibits growth, promotes apoptosis	PPAR-α	[83]
miR-27a-3p	GCs	PCOS	inhibits proliferation, promotes apoptosis	SMAD5	[84]
miR-6881-3p	GCs	DOR	diminishes viability, promotes apoptosis, regulates steroidogenesis	SMAD4	[85]
miR-106a	serum, GCs	DOR	increases viability, attenuates apoptosis	ASK1	[86]
miR-122-5p	ovarian-derived exosomes	POI	promotes apoptosis	BCL9	[90]
miR-26b	adipocytes-derived extracellular vesicles	PCOS	promotes CC apoptosis, inhibits CC proliferation	-	[91]
miR-133a	GCs	-	promotes apoptosis	-	[93]
miR-194	GCs	PCOS	suppresses growth, induces apoptosis	HB-EGF	[95]
miR-128-3p	KGN cells	-	inhibits proliferation, promotes apoptosis	GHSR	[96]
miR-130b-3p	(luteinized) GCs	PCOS	promotes proliferation, regulates cell cycle, inhibits apoptosis	SMAD4	[98]
miR-126-3p	-	POF	promotes proliferation, attenuates apoptosis	PIK3R2	[104]
miR-let-7d-3p	GCs	PCOS	inhibits proliferation	TLR4	[107]
miR-646	KGN cells	PCOS	inhibits proliferation and viability, induces apoptosis and G2/M process	IGF-1	[116]
miR-423-5p	KGN cells	high ovarian response	(downregulation) increases the number of cells in the S phase and the concentration of E_2_	CSF1	[125]
miR-96-5p	serum, GCs, follicle fluid	PCOS	stimulates E_2_ synthesis, promotes proliferation, inhibits apoptosis, increases the proportion of S phase cells, reduces MMP depolarization	FOXO1	[126]
miR-21	hucMSC-derived exosomes	-	promotes E_2_ secretion	LATS1	[127]
miR-18a-3p, miR-20b-5p	plasma exosomes	PCOS	inhibits E_2_ synthesis	-	[128]
miR-126-3p	plasma exosomes	PCOS	inhibits proliferation and progesterone synthesis, promotes E_2_ synthesis	PDGFRβ	[128]
miR-146a-5p	plasma exosomes	PCOS	inhibits proliferation and progesterone synthesis, promotes E_2_ synthesis	-	[128]
miR-106a-5p	plasma exosomes	PCOS	inhibits E_2_ synthesis, promotes progesterone synthesis	-	[128]
miR-484	SVOG cells; GCs	-; DOR	induces mitochondrial depolarization, increasing apoptosis; induces mitochondrial dysfunction and oxidative stress, reduces viability, promotes apoptosis, increases the cells in G0/G1 phase	SESN2; YAP1	[133]; [134]
miR-125b-5p miR-132-3p miR-19a-3p miR-30a-5p miR-660-5p	follicle fluid	DOR	regulates mitochondrial proteins and biogenesis, controls metabolism	-	[137]
miR-133a-3p	GCs	PCOS	induces insulin resistance	PI3K/AKT	[138]
miRNA-146	GCs	POI	attenuates inflammatory response and oxidative stress, relieves apoptosis, enhances viability	TLR4/NF-κB	[140]
miR-424-5p	follicular fluid derived exosomes, GCs	PCOS	promotes senescence, inhibits proliferation, increases cell number in G1 phase	CDCA4	[143]

**Table 2:** Regulation functions of lncRNAs in GCs

Name	Sources	Related diseases	Effects on GCs	Target genes	References
lncRNA FMR6	follicle fluid, GCs	POF	promotes apoptosis, inhibits proliferation	SAV1	[82]
lncRNA PVT1	GCs	POI	(downregulation) promotes apoptosis	Foxo3a	[87]
lncRNA DLEU1	GCs	POF	increases apoptosis	miR-146b-5p	[88]
lncRNA XIST	KGN cells	PCOS	inhibits viability, induces apoptosis	miR-30c-5p/Bcl2L11	[89]
linc00092	follicular fluid-derived small extracellular vesicles	PCOS	alleviates apoptosis	miR-18b-5p	[92]
lncRNA PWRN2	serum, GCs	PCOS	(knockdown) enhances viability, inhibits apoptosis	ATRX	[97]
linc00173	GCs	PCOS	inhibits proliferation, promotes apoptosis	miR-124-3p/JAG1	[99]
lncRNA SNHG12	KGN cells, COV434 cells, SVOG cells	PCOS	promotes proliferation, inhibits apoptosis	miR-129, miR-125b	[101]
lncRNA HCP5	hucMSC-derived exosomes; KGN cells	-	promotes proliferation, inhibits apoptosis; (downregulation) induces G1 phase arrest and apoptosis, inhibits proliferation	MSI2/ESR1; miR-27a-3p/IGF-1	[103]; [111]
lncRNA SNHG5	GCs	PCOS	inhibits proliferation, induces apoptosis and G0/G1 phase arrest	miR-92a-3p/CDKN1C	[109]
lncRNA PWRN1	GCs, oocytes	DOR, PCOS	(downregulation) induces S phase arrest, apoptosis and autophagy, inhibits steroidogenesis	-	[112]
lncRNA GCAT1	GCs	POI	(downregulation) induces G1/S phase arrest, inhibits proliferation	PTBP1/p27	[114]
lnc-GULP1-2:1	luteinized GCs	DOR, PCOS	inhibits proliferation, induces G1/S phase arrest	-	[115]
lncRNA HOTAIR	KGN cells; GCs; GCs at logarithmic growth phase	POI; PCOS; PCOS	promotes proliferation and autophagy; (downregulation) inhibits apoptosis, elevates viability; inhibits proliferation, enhances apoptosis	miR-148b-3p/ATG14; IGF-1/PI3K/AKT; miR-130a/IGF-1	[119]; [120]; [121]
lncRNA MEG3	GCs	PCOS	induces autophagy, inhibits proliferation; (silencing) repairs MMP, increasing proliferation and viability, inhibits apoptosis	PI3K/AKT/mTOR; miR-21-3p	[122]; [136]
lncRNA NEAT1	GCs	POF	inhibits apoptosis and autophagy	miR-654/STC2	[123]
lncRNA LIPE-AS1	follicular fluid-derived exosomes	PCOS	upregulates steroidogenesis-related gene expression, promotes proliferation, decreases apoptosis	miR-4306	[124]
lnc-CCNL1-3:1	luteinized GCs	PCOS	promotes apoptosis, suppresses mitochondrial function, suppresses transportation and uptake of glucose, induces insulin resistance	FOXO1	[135]
lncRNA ZNF674-AS1	GCs	POI	(silencing) inhibits proliferation, suppresses glucose metabolism	ALDOA	[139]
lncRNA AOC4P	GCs	PCOS	promotes proliferation, inhibits apoptosis, reduces pro-inflammatory cytokines	NF-κB	[141]
lncRNA DANCR	GCs	POI	(knockdown) induces senescence and G1 phase arrest, inhibits proliferation	hNRNPC	[142]

**Table 3:** Regulation functions of circrnas in GCs

Name	Sources	Related diseases	Effects on GCs	Target genes	References
circ_0043532	GCs	PCOS	(knockdown) suppresses proliferation and cell cycle process, promotes apoptosis	miR-182/SGK3	[100]
circ_RANBP9	plasma, KGN cells, COV434 cells	PCOS	(silence) inhibits proliferation, accelerates apoptosis	miR-136-5p/XIAP	[102]
circ-FURIN	CCs; KGN cells	PCOS	(knockdown) suppresses proliferation, facilitates apoptosis; (depletion) promotes proliferation, represses apoptosis (of KGN cells induced by TTR)	miR-195-5p/BCL2; miR-423-5p/MTM1	[105]; [106]
circ_0002021	hucMSCs-derived exosomes	POF	mitigates G0/G1 phase arrest, oxidative stress injury and senescence	miR-125a-5p/CDK6	[110]
circ_BECN1	GCs	PCOS	(downregulation) inhibits proliferation, promotes apoptosis, induces G1/S phase arrest	miR-619-5p/Rab5b	[113]
circRHBG	GCs	PCOS	(knockdown) suppresses proliferation, promotes ferroptosis	miR-515/SLC7A11	[144]

### ncRNAs Regulate Apoptosis in GCs

Apoptosis is a process where a cell ceases to grow and divide, ultimately leading to its controlled death without releasing its contents into the surrounding environment. This process, often termed programmed cell death or “cell suicide”, plays a critical role in various biological functions [78].

During follicular development, GC apoptosis is a common occurrence. While moderate GC apoptosis is essential for normal follicular development [79], aiding in the regulation of follicular atresia [80] and the selection of dominant follicles [81], excessive or insufficient apoptosis can negatively impact follicular development.

Increasing research has focused on the regulation of GC apoptosis by ncRNAs. Systematic studies employing diverse methodologies, including clinical trials, in vitro experiments, in vivo experiments, and rescue experiments, have provided insights into the role of ncRNAs in GC apoptosis. For instance, lncRNA Fragile X Mental Retardation 6 (FMR6) has been shown to promote GC apoptosis by binding to Salvador family WW domain containing protein 1 (SAV1), while its silencing can inhibit apoptosis and enhance cell proliferation [82]. Similarly, miR-4433a-3p [83], miR-27a-3p [84], and miR-6881-3p [85] have been identified as promoters of GC apoptosis. Conversely, certain ncRNAs, such as miR-106a, inhibit GC apoptosis. Downregulation of miR-106a can promote apoptosis by upregulating apoptosis signal-regulating kinase 1 (ASK1), whereas miR-106a itself enhances GC vitality [86]. lncRNA plasmacytoma variant translocation 1 (PVT1) has also demonstrated an anti-apoptotic effect in in-vivo studies; its overexpression in mouse ovaries reversed cell apoptosis, potentially through the downregulation of forkhead box (FOX)O3a (FOXO3a) [87].

lncRNAs can further influence GC apoptosis by sponging miRNAs, forming regulatory networks. For example, lncRNA deleted in lymphocytic leukemia 1 (DLEU1) promotes granulosa-like tumor cell line (KGN) apoptosis by sponging miR-146b-5p [88], while lncRNA X-inactive specific transcript (XIST) induces KGN cells apoptosis *via* the miR-30c-5p/Bcl2-like protein 11 signaling axis [89].

Additionally, ncRNAs derived from sources outside GCs can be transported to GCs through extracellular vesicles or exosomes, influencing apoptosis. For instance, miR-122-5p in ovarian-derived exosomes and miR-26b in mature adipocyte-derived extracellular vesicles promote GC and CC apoptosis [90, 91]. Conversely, linc00092 delivered by follicular fluid-derived extracellular vesicles has been found to mitigate dehydroepiandrosterone (DHEA)-induced GC apoptosis, thereby exerting a protective effect [92]. These results underscore the diverse origins of regulators involved in GC apoptosis.

Furthermore, certain miRNAs, such as miR-133a, require further investigation to fully understand their role in apoptosis regulation. While miR-133a has consistently been observed to enhance GC apoptosis in animal and in vitro studies, bioinformatics analyses suggested that it may target both pro-apoptotic and anti-apoptotic genes [93], indicating a complex regulatory role. Thus, miR-133a might maintain a balance between GC survival and death through multiple targets, though additional in vivo and in vitro studies are necessary to elucidate its precise effects.

### ncRNAs Regulate Proliferation in GCs

GCs surround the oocyte and create a vital microenvironment necessary for oocyte maturation during their own proliferation, influencing the overall development and maturation of the follicle [94]. The health of the follicle is intricately tied to the balance between GC proliferation and apoptosis; any disruption in this balance can lead to follicular atresia [81].

Extensive research has focused on the regulatory roles of ncRNAs in GC proliferation, often revealing dual roles of these molecules in influencing both proliferation and apoptosis. For instance, the downregulation of miR-194 enhances GC proliferation, whereas its high expression triggers apoptosis [95]. Similarly, miR-128-3p and lncRNA Prader-Willi region nonprotein coding RNA (PWRN) 2 are known to inhibit GC proliferation while promoting apoptosis [96, 97]. Conversely, miR-130b-3p exhibits the opposite effect, fostering GC proliferation and reducing apoptosis [98].

Some studies have delved into the competitive endogenous RNA (ceRNA) axis, which involves interactions between lncRNAs, circRNAs, and downstream miRNAs, in regulating GC proliferation. For example, linc00173 inhibits GC proliferation and induces apoptosis *via* the miR-124-3p/jagged canonical Notch ligand 1 (JAG1) pathway, with in vivo experiments showing that linc00173 downregulation can normalize hormone levels in PCOS rats [99]. On the other hand, circ_0043532 knockdown inhibits GC proliferation and cell cycle progression and promotes apoptosis by regulating the miR-182/serum/glucocorticoid regulated kinase family member 3 (SGK3) axis [100]. Silencing lncRNA small nucleolar RNA host gene (SNHG) 12 and circ_RANBP9 in GCs inhibits cell viability and proliferation while promoting apoptosis through sponging miR-129 and miR-125b, and miR-136-5p/X-linked inhibitor of apoptosis protein (XIAP) pathway, respectively [101, 102].

Moreover, exosomes derived from human umbilical cord mesenchymal stem cells (hucMSCs) contain ncRNAs that regulate GC proliferations. For instance, overexpression of hucMSCs-exosome-derived lncRNA human leukocyte antigen complex P5 (HCP5) promotes GC proliferation and inhibits apoptosis by regulating the musashi RNA-binding protein 2/estrogen receptor alpha 1 (MSI2/ESR1) axis [103]. Similarly, miR-126-3p delivered by hucMSCs-exosomes has been shown to enhance proliferation and inhibit apoptosis in GCs damaged by cisplatin [104].

However, the regulatory role of certain ncRNAs in GC proliferation is subject to debate. For example, circ-FURIN’s deletion has been reported to inhibit proliferation and promote apoptosis through the miR-195-5p/B-cell lymphoma/leukemia-2 (BCL-2) pathway [105]. In contrast, in testosterone (TTR)-induced KGN cells, circ-FURIN depletion alleviated proliferation inhibition and apoptosis, suggesting a role in promoting proliferation and inhibiting apoptosis [106]. These contradictory findings may stem from differences in the experimental conditions, such as the states of KGN cells used (TTR-induced [106] versus no modeling treatment [105]). It is also essential to rule out the influences of regulators that downregulate circ-FURIN expression, as demonstrated in studies where KGN cells were transfected with small interfering RNA (si)-circ-FURIN in one instance [105] and small hairpin RNA (sh)-circ-FURIN in another [106].

Some ncRNAs, like miR-let-7d-3p, have preliminarily shown inhibitory effects on GC proliferation in vitro [107], but their influence on other GC phenotypes and their in vivo effects warrant further investigation.

### ncRNAs Regulate the Cell Cycle in GCs

Cell growth and proliferation are intricately linked to the orderly regulation of the cell cycle, which includes interphase and the mitotic phase (M phase). Interphase is composed the G1, S, and G2 phases. The G1 phase is a preparatory stage for DNA synthesis, involving RNA and protein synthesis, while the S phase is dedicated to DNA replication. The G2 phase primarily involves the synthesis of protein in preparation for entering the M phase. During the G1 phase, many cells in the adult body reside in a resting, non-proliferative state known as the G0 phase, and must transition back to the G1 phase to re-enter the cell cycle [108]. 

The cell cycle of GCs is regulated by various ncRNAs that target and influence different phases. For instance, during the G0/G1 phase, GCs may be arrested due to the abnormal overexpression of lncRNA SNHG5, which affects DNA replication and repair pathways through the lncRNA SNHG5/miR-92a-3p/cyclin dependent kinase inhibitor 1 C (CDKN1C) axis [109]. Additionally, circ_0002021, derived from hucMSCs exosomes, could alleviate cyclophosphamide (CTX)-induced oxidative stress in GCs, reduce G0/G1 phase arrest, and mitigate cellular senescence by competitively sponging miR-125a-5p and upregulating cyclin-dependent kinase (CDK) 6 [110]. In the G1 phase, the cell cycle can be stalled due to the downregulation of lncRNA HCP5 *via* the lncRNA HCP5/miR-27a-3p/insulin-like growth factor (IGF)-1 axis [111]. Conversely, downregulation of lncRNA PWRN1 accelerates the G1/S transition by increasing the expression of cell-cycle related proteins CDK4 and cyclin D1, reducing the number of GCs in the G1 phase, increasing those in the S phase, and causing cell development to stagnate in the S phase. This downregulation also promotes apoptosis and autophagy while reducing steroidogenesis [112]. GCs may also experience G1/S arrest due to the silencing or downregulation of circ_Beclin1 (BECN1) [113], lncRNA granulosa cell-associated transcript 1 (GCAT1) [114], or be inhibited from progressing from G1 to S phase by overexpression of lnc-GULP1-2:1 [115]. During the S phase, GCs can be arrested if miR-646 is downregulated, while upregulation of miR-646 promotes the transition to the G2/M phase [116].

Abnormalities in the cell cycle are often associated with irregularities in GC apoptosis and proliferation. For example, lncRNA PWRN1 [112] can simultaneously affect the cell cycle and apoptosis, as can lncRNA SNHG5 [109], circ_BECN1 [113], miR-646 [116]. Additionally, several ncRNAs, such as lncRNA SNHG5 [109], lncRNA HCP5 [111], circ_BECN1 [113], lncGCAT1 [114], lnc-GULP1-2:1 [115], regulate both the cell cycle and cell proliferation. Additionally, some previously mentioned ncRNAs that influence GC proliferation and apoptosis also impact the cell cycle, such as miR-130b-3p [98], which promotes GC proliferation and induces S-phase arrest, and circ_0043532 [100], whose downregulation inhibits proliferation, cell cycle progression, and promotes apoptosis in PCOS GCs.

### ncRNAs Regulate Autophagy in GCs

Autophagy is a cellular process wherein lysosomes degrade dysfunctional or excessive cytoplasmic components, recycling the resulting degradation products to synthesize new proteins and organelles or provide energy [117]. This mechanism allows cells to accelerate their metabolic cycle and adapt to nutrient-deprived environments, serving as a survival strategy. Additionally, autophagy plays a critical role in promoting programmed cell death [118].

ncRNAs are key regulators of autophagy in GCs, a process closely linked to various physiological events such as proliferation, apoptosis, and cell cycle progression, thereby influencing follicular development and ovarian function [13]. In some cases, autophagy positively impacts the growth and development of GCs and maintains ovarian function. For example, the overexpression of lncRNA HOX transcript antisense intergenic RNA (HOTAIR) in a cisplatin-induced POI model increases autophagy levels in GCs, promoting cell proliferation through the miR-148b-3p/autophagy-related gene 14 (ATG14)-mediated autophagy pathway [119]. However, in the context of PCOS, lncRNA HOTAIR has been found to inhibit proliferation and promote apoptosis in GCs [120, 121]. This complexity suggests that lncRNA HOTAIR’s regulation effects on GC survival and apoptosis may depend on the specific pathological state of the cells. Further research is needed to fully understand the interplay between autophagy induced by lncRNA HOTAIR and its impact on GC apoptosis and proliferation.

Moreover, several studies have shown that ncRNA-mediated upregulation of autophagy in GCs often promotes apoptosis and inhibits proliferation. For instance, as mentioned earlier, downregulation of lncRNA PWRN1 not only affects the G1/S transition but also upregulates autophagy-related genes microtubule-associated protein 1 light chain (LC)3 A and LC3B, leading to increased autophagy levels that inhibit proliferation and promote apoptosis [112]. Similarly, high expression of lncRNA maternally expressed gene 3 (MEG3) in GCs from PCOS rats induces autophagy, resulting in reduced proliferation and cell number [122]. Additionally, overexpression of lncRNA nuclear enriched abundant transcript 1 (NEAT1), through the regulation of the miR-654/stanniocalcin-2 (STC2)/mitogen-activated protein kinase (MAPK) axis, can reduce both autophagy and apoptosis levels in CTX-treated GCs, further highlighting the dual regulatory role of ncRNAs in GC autophagy and apoptosis [123].

### ncRNAs Regulate Steroidogenesis in GCs

Steroidogenesis is a critical function of ovarian GCs, influencing follicular development through the secretion of steroid hormones [112]. Any abnormalities in steroid production or metabolism can alter the follicular fluid’s local environment, directly or indirectly affecting follicular growth and function [124].

The impact of ncRNAs on GCs and ovarian function can be mediated through their regulation of steroidogenesis. For instance, miR-6881-3p affects GCs not only by promoting apoptosis but also by modulating the expression of gonadotropin receptors (follicle stimulating hormone receptor (FSHR), luteinizing hormone/choriogonadotropin receptor (LHCGR)) and steroid hormone-generating enzymes, such as cholesterol side-chain lyase (cytochrome P450 family 11 subfamily A member 1, CYP11A1). It influences steroidogenesis by downregulating FSHR and CYP11A1 mRNA levels while upregulating LHCGR mRNA [85]. Similarly, studies on lncRNA PWRN1 have shown that its downregulation not only affects GC apoptosis, cell cycle progression, and autophagy, but also inhibits the production of estradiol (E_2_) and progesterone [112]. Additionally, miR-423-5p has been found to promote E_2_ secretion in its downregulated state, accompanied by a higher number of S-phase cells [125]. On the other hand, miR-96-5p targets and downregulates FOXO1, thereby regulating the expression of downstream genes like cytochrome P450 family 19 subfamily A member 1 (CYP19A1) and steroidogenic factor (SF)1, which promotes E_2_ synthesis, enhances cell proliferation, and inhibits GC apoptosis [126].

ncRNAs from sources other than GCs can also influence steroidogenesis in GCs. For example, follicular fluid-derived exosomal lncRNA LIPE antisense RNA 1 (LIPE-AS1) can target and sponge miR-4306 in GCs, promoting the expression of steroid hormone synthesis-related genes such as steroidogenic acute regulatory protein (StAR), CYP11A1, and cytochrome P450 family 17 subfamily A member 1 (CYP17A1), and increase E_2_ level. This action facilitates GC proliferation and prevents apoptosis in KGN cells, ultimately affecting oocyte development and maturation [124]. Similarly, miR-21 carried by hucMSCs-derived exosomes downregulates large tumor suppressor 1 (LATS1) in GCs, reducing phosphorylated Lysyl oxidase like 2 (LOXL2) and Yes-associated protein (YAP) levels, which in turn promotes estrogen secretion [127]. Furthermore, plasma exosomes from patients with PCOS contain various differentially expressed miRNAs that exert complex effects on steroidogenesis and GC proliferation. For example, the overexpression of miR-18a-3p, miR-20b-5p, and miR-106a-5p significantly inhibits E_2_ synthesis, while miR-106a-5p overexpression also significantly promotes progesterone synthesis. In contrast, the overexpression of miR-126-3p and miR-146a-5p significantly enhances E_2_ synthesis while inhibiting progesterone secretion and GC proliferation [128].

### ncRNAs Regulate Mitochondrial Function in GCs

Mitochondrial function is essential for GC function and follicular development [129], and disruptions in mitochondrial activity are associated with ovarian dysfunction [130, 131]. Recent studies have begun to elucidate the mechanisms by which ncRNAs regulate key GC processes, such as proliferation and apoptosis, through their influence on mitochondrial dynamics. One notable example is miR-484, which plays a significant role in mitochondrial fission and fusion [132]. Overexpression of miR-484 in GCs has been shown to increase mitochondrial fragmentation, contribute to mitochondrial membrane potential (MMP) depolarization and promote mitochondrial apoptosis [133]. Further research identifies that linc00958, acting as a ceRNA for miR-484, forms a signaling axis with Sestrin2 (SESN2) under oxidative stress (referred to as the linc00958/miR-484/SESN2 axis). This axis regulates mitochondrial dysfunction and mitochondria-associated apoptosis in GCs [133]. Additionally, miR-484 has been found to directly target YAP1 mRNA in GCs, leading to mitochondrial dysfunction, increased MMP depolarization, reduced mitochondrial reactive oxygen species (ROS) scavenging, and decreased adenosine triphosphate (ATP) synthesis, ultimately lowering GC viability and promoting apoptosis [134]. In KGN cells, miR-96-5p overexpression has been associated with increased MMP, enhanced cell viability, and reduced apoptosis rates, suggesting that miR-96-5p supports GC proliferation and inhibits apoptosis through regulation of the mitochondrial apoptotic pathway [126]. Conversely, lnc-CCNL1-3:1 overexpression has been shown to increase ROS production, decrease ATP levels, and impair mitochondrial function in GCs, leading to cell apoptosis [135]. Silencing lncRNA MEG3 can repair MMP and reduce expression of apoptotic proteins in the early stages of GC apoptosis, thereby enhancing cell viability and reducing apoptosis [136].

While research on the role of ncRNAs in regulating mitochondrial function in GCs is still in its early stages, some preliminary studies offer promising insights. For instance, differentially expressed miRNAs in the follicular fluid of women with poor ovarian reserve—such as miR-125b-5p, miR-132-3p, miR-19a-3p, miR-30a-5p, and miR-660-5p—have been implicated in the regulation of mitochondrial proteins and biogenesis [137]. These findings suggest potential avenues for further exploration into the mechanisms by which ncRNAs modulate mitochondrial function in GCs, offering new perspectives on ovarian health and dysfunction.

### Other Regulatory Effects of ncRNAs on GCs

ncRNAs significantly impact GCs by modulating energy metabolism, inflammatory responses, cellular aging, and more. Upregulation of lncRNA CCNL1-3:1 not only impairs mitochondrial function but also disrupts glycometabolism in GCs by enhancing FOXO1 expression. This disruption leads to reduced glucose transport, inhibited glucose uptake, induced insulin resistance, and increased GC apoptosis and negatively affects follicular development [135]. Similarly, miR-133a-3p downregulates phosphoinositide 3-kinase/protein kinase B (PI3K/AKT) signaling in GCs, altering the expression of glucose metabolism-related proteins downstream of this pathway, thereby inducing insulin resistance in ovarian GCs [138], a condition closely linked to the pathogenesis of PCOS. Downregulation of lncRNA ZNF674 antisense RNA 1 (ZNF674-AS1), an energy stress-responsive lncRNA, reduces the aldolase A (ALDOA) activity, inhibiting glucose metabolism in GCs, lowering levels of glycolytic intermediates and ATP, and ultimately impairing cell proliferation [139].

In the context of inflammatory responses, miRNA-146 has been shown to inhibit the Toll-like receptor 4 (TLR4) /nuclear factor kappa B (NF-κB) signaling pathway, downregulate tumor necrosis factor-α (TNF-α) and interleukin-6 (IL-6) expression, thereby reducing inflammation and oxidative stress, promoting GC viability, and alleviating GC apoptosis [140]. lncRNA amine oxidase coppercontaining 4 pseudogene (AOC4P) exerts similar anti-inflammatory effects by inhibiting NF-κB pathway activation, which promotes GC proliferation and reduces apoptosis [141].

Regarding cellular aging, ncRNAs also play a pivotal role. For instance, lncRNA Differentiation antagonizing non-protein coding RNA (DANCR) negatively regulates GC senescence and follicular atresia [142]. Its downregulation diminishes the interaction between heterogeneous nuclear ribonucleoprotein C (hNRNPC) and p53, leading to increased expression of p53 and p21, which accelerates GC aging, inhibits proliferation, induces cell cycle arrest, and causes DNA damage, ultimately resulting in follicle atresia. Conversely, miR-424-5p, derived from follicular fluid exosomes, significantly promotes GC senescence and suppresses GC proliferation by blocking the cell division cycle associated 4 (CDCA4)-mediated retinoblastoma protein/E2F transcription factor 1 (Rb/E2F1) signaling pathway and upregulating of senescence-associated genes [143].

Recent research has also explored the role of ferroptosis, a newly identified form of cell death, in GCs. Downregulation of circRHBG has been found to promote ferroptosis through the circRHBG/miR-515-5p/solute carrier family 7 member 11 (SLC7A11) axis, thereby inhibiting GC proliferation [144]. This emerging area warrants further investigation, as it offers new insights into GC regulation and ovarian function.

### piRNAs in Ovarian GCs

Unlike miRNAs, lncRNAs, and circRNAs, the influence of piRNAs on ovarian function remains relatively underexplored. Most studies have concentrated on the regulatory roles of piRNAs in oocyte development, gene expression, and transposon silencing, with research primarily conducted on *Drosophila* [40, 41] and a limited number of mammals [45, 145, 146]. Some investigations have explored piRNA expression in ovarian somatic cells, including GCs (or follicular cells), and their impact on germline cell differentiation, revealing distinct piRNA pathways in somatic cells compared to germ cells [40, 41, 147–150]. One current perspective suggests that piRNAs are abundant in the fetal human ovaries but deficient in adult ovaries and absent in somatic tissues [151]. However, another study has identified piRNAs in the CCs surrounding human oocytes, noting variability in their presence associated with different disease states [45]. These findings highlight the need for further research to elucidate the specific regulatory roles of piRNAs in follicle development and GC function in mammals, particularly in humans.

## The Impact of GC-related ncRNAs on Ovarian Dysfunction

### Polycystic Ovary Syndrome (PCOS)

**Fig. 4:**
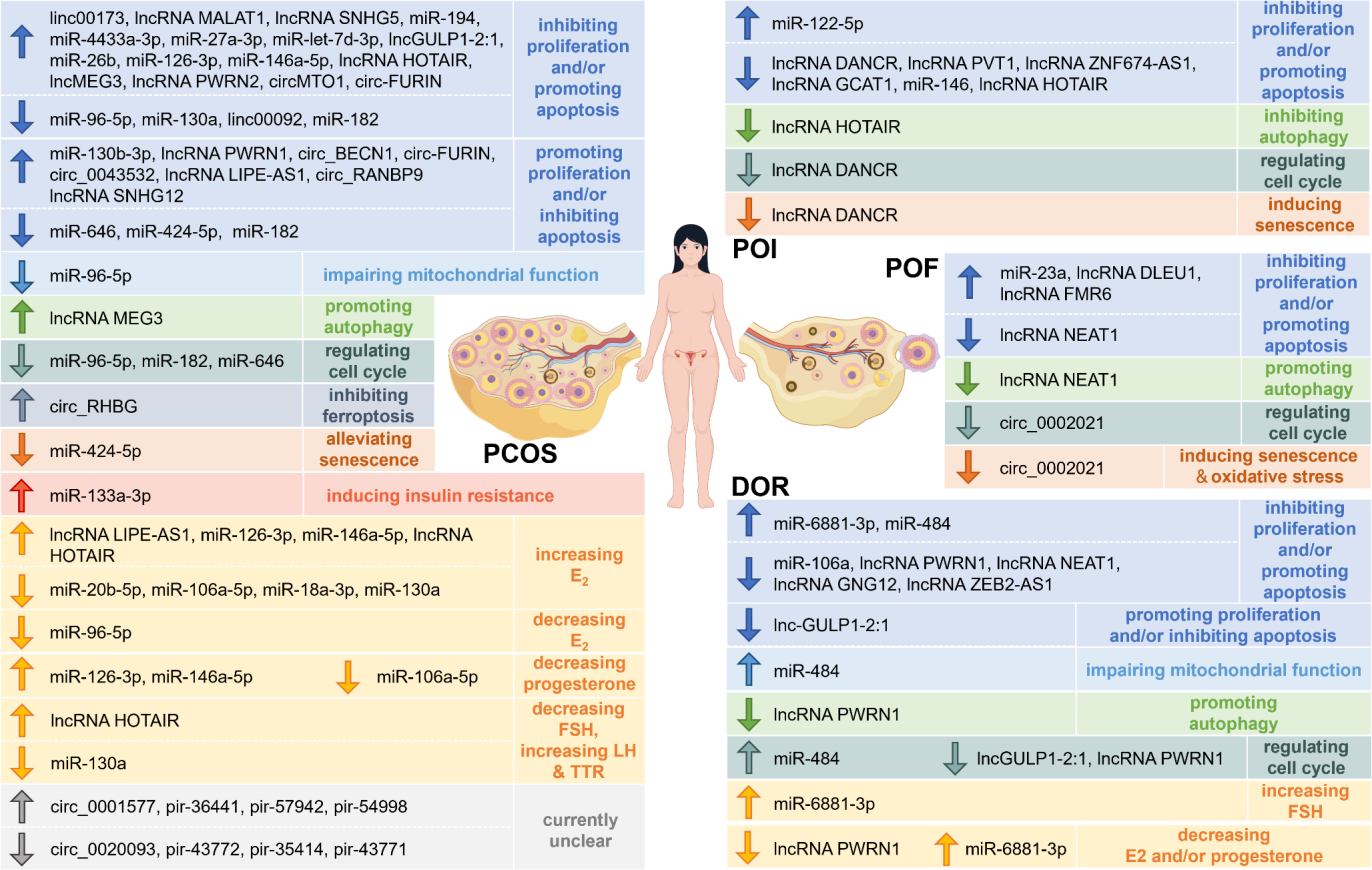
Differentially expressed ncRNAs of functional importance in PCOS, POF, POI, DOR. ncRNAs exhibit different expression trends in various ovarian dysfunctions. Their upregulation or downregulation can participate in the pathogenesis of PCOS, POF, POI, and DOR by differentially affecting the proliferation, apoptosis, cell cycle, autophagy, mitochondrial function, steroidogenesis, cellular senescence, ferroptosis, glycometabolism, and oxidative stress of GCs. The figure was drawn with Figdraw

**Fig. 5:**
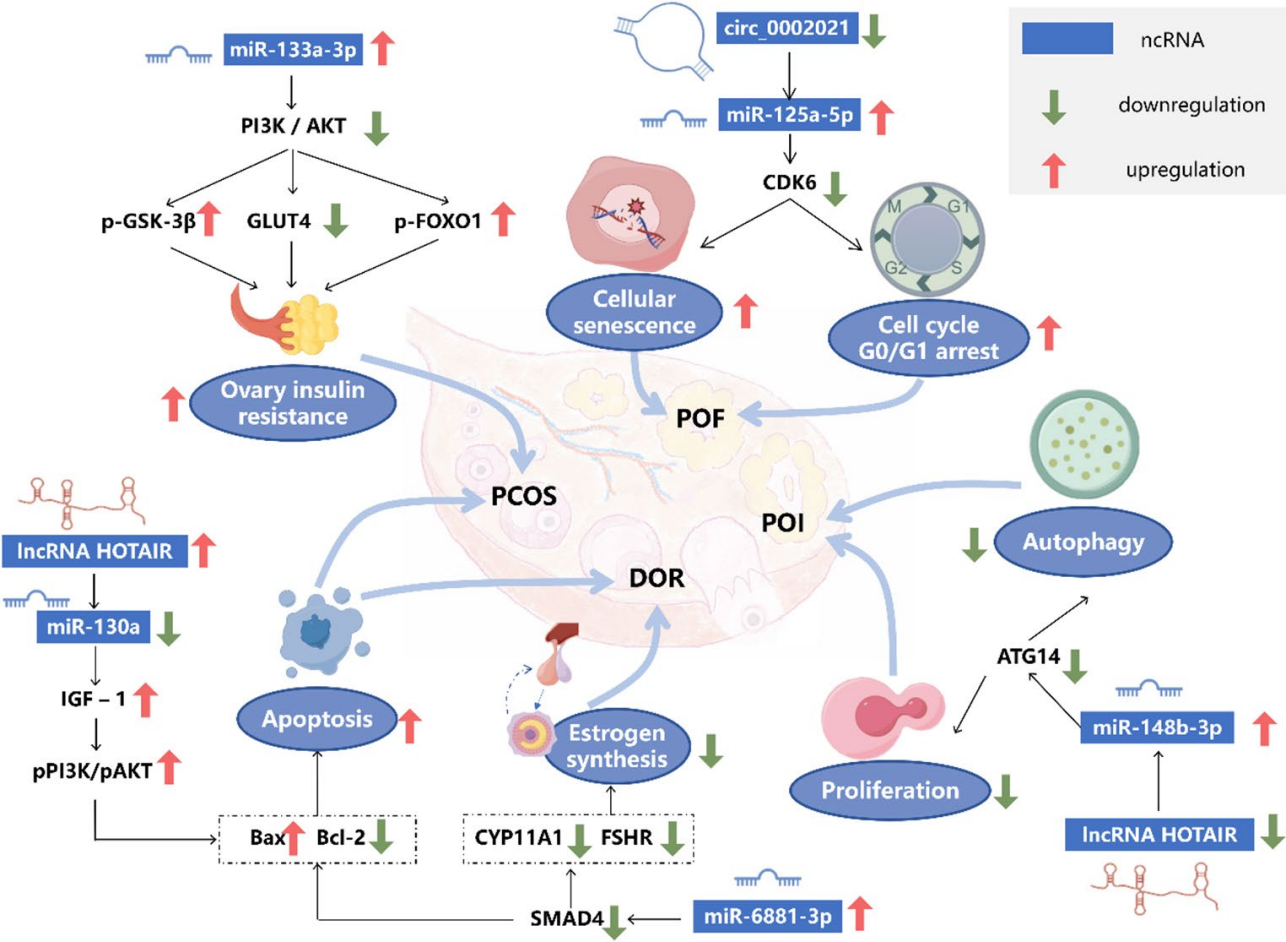
Hypothetical diagram of the mechanism by which ncRNAs regulate GCs involved in ovarian dysfunction. ncRNAs can participate in the pathogenesis of ovarian dysfunctions by influencing various life activities and cellular phenotypes of GCs. A single ovarian dysfunction may have multiple pathogenic mechanisms, regulated by different ncRNAs. For instance, the development of PCOS can be jointly regulated by miR-133a-3p and lncRNA HOTAIR. The same ncRNA can be involved in the pathogenesis of the same disease through different pathways, such as miR-6881-3p contributing to DOR by increasing apoptosis and reducing estrogen synthesis, and the downregulation of circ_0002021 leading to POF by inducing senescence and cell cycle arrest. Some ncRNAs can be involved in the regulation of different diseases, such as lncRNA HOTAIR, which when upregulated can cause PCOS by increasing GC apoptosis, and when downregulated can induce POI by reducing proliferation and autophagy. The figure was drawn with Figdraw

PCOS is the most prevalent reproductive endocrine disorder in women of reproductive age, characterized by hyperandrogenemia, polycystic ovarian morphology, and infrequent ovulation, which often leads to irregular menstruation [152]. Additionally, PCOS is associated with various metabolic complications, including insulin resistance, obesity, hyperlipidemia, type 2 diabetes mellitus, and an increased risk of cardiovascular diseases and psychological disorders [153]. Furthermore, PCOS heightens the risk of several pregnancy-related complications, such as premature delivery, gestational diabetes, pregnancy-induced hypertension, and pre-eclampsia, all of which can severely impact women’s reproductive health [154]. Consequently, the pathogenesis of PCOS has garnered significant attention. Research has shown that PCOS is related to follicular arrest [152, 155], with the imbalance of GC proliferation and apoptosis being a key factor in abnormal follicular development. GC dysfunctions, such as abnormal proliferation and apoptosis, abnormal steroidogenesis, and ovarian insulin resistance, is often observed in PCOS cases [156] (Fig. 4). These GC dysfunctions are associated with the regulation of various ncRNAs, reflecting the diversity and complexity of the role of ncRNAs in the pathogenesis of PCOS (Fig. 5).

As previously discussed, numerous studies have confirmed that ncRNAs regulate various GC activities, including proliferation and apoptosis, and some ncRNAs are closely linked to PCOS. In the GCs of patients with PCOS, increased expression of linc00173 [99], lncRNA metastasis-associated lung adenocarcinoma transcript 1 (MALAT1) [157], lncRNA SNHG5 [109], miR-4433a-3p [83], miR-27a-3p [84], miR-194 [95], miR-let-7d-3p [107] has been observed, which contributes to the inhibition of proliferation and/or the promotion of apoptosis. Similarly, lncGULP1-2:1 [115] has shown comparable regulatory effects in luteinized GCs of patients with PCOS. On the other hand, although miR-130b-3p [98], circ_BECN1 [113], and circ_0043532 [100] are also highly expressed in GCs of patients with PCOS, they promote proliferation and/or inhibit apoptosis. Additionally, lncRNA PWRN1 is upregulated in PCOS GCs, and its deletion leads to increased apoptosis [112]. circRHBG, significantly upregulated in PCOS GCs, inhibits ferroptosis in these cells through the circRHBG/miR-515-5p/SLC7A11 axis [144]. Moreover, miR-133a-3p (Fig. 5), highly expressed in GCs from obese patients with PCOS, plays a critical role in promoting ovarian insulin resistance, which is closely associated with PCOS pathogenesis [138].

There are also ncRNAs downregulation in GCs of patients with PCOS. For example, the downregulation of miR-646 may promote GC proliferation through the IGF-1 pathway [116]. Conversely, lncRNA AOC4P, which normally promotes proliferation and inhibits apoptosis, has reduced effects due to its downregulation in patients with PCOS [141]. Preliminary studies have also identified that circ_0001577 is upregulated and circ_0020093 is downregulated in PCOS GCs [158], and though their specific regulatory effects on GCs require further investigation.

In patients with PCOS, the expression of lncRNA LIPE-AS1 in follicular fluid-derived exosomes is increased, which facilitates GC proliferation, inhibits apoptosis, and enhances steroid hormones synthesis, thereby exacerbating PCOS [124]. In the plasma of patients with PCOS, circ_RANBP9 is upregulated, and its silencing inhibits proliferation and promotes apoptosis in GCs by targeting the miR-136-5p/XIAP pathway [102]. Additionally, extracellular vesicles derived from mature adipocytes present in the serum of patients with PCOS contain elevated levels of miR-26b, which, according to in vitro studies, inhibits GC viability and increases apoptosis [91]. Research on plasma exosomes in patients with PCOS has also shown that miR-126-3p and miR-146a-5p are significantly upregulated, promoting E_2_ synthesis, inhibiting progesterone synthesis, and suppressing GC proliferation. Conversely, miR-20b-5p, miR-106a-5p, and miR-18a-3p are significantly downregulated and inhibit E_2_ synthesis when overexpressed [128, 159]. In patients with patients, miR-96-5p is downregulated in serum, follicular fluid, and GCs, resulting in inhibited E_2_ secretion and reduced GC proliferation [126]. Furthermore, miR-424-5p from follicular fluid-derived exosomes is significantly reduced in patients with PCOS, and its downregulation promotes GC proliferation and alleviates GC senescence [143].

Animal experiments have demonstrated that lncRNA HOTAIR and lncRNA MEG3 are significantly upregulated in ovaries and GCs of PCOS rats. The knockdown or silencing of these molecules has been shown to enhance GC proliferation and reduce apoptosis [120–122, 136]. Similarly, the highly expressed lncRNA PWRN2 in the ovaries and serum of PCOS rats also boosts GC vitality and inhibits apoptosis when downregulated [97]. In contrast, although lncRNA SNHG12 is also upregulated in the ovarian tissues of PCOS rats, its silencing diminishes GC vitality and proliferation while promoting apoptosis [101].

In vitro studies have revealed a significant reduction in linc00092 levels in DHEA-induced PCOS models of KGN cells, whereas linc00092 in follicular fluid-derived extracellular vesicles mitigates DHEA-induced cellular damage [92]. circular RNA-mitochondrial tRNA translation optimization 1 (circMTO1) is notably upregulated in a PCOS model of human granulosa-like tumor cells induced by insulin, and its knockdown has been observed to promote proliferation and inhibit apoptosis [160].

The differential expression of piRNAs in patients with PCOS has also been preliminarily explored, identifying several significantly upregulated piRNAs (e.g., pir-36441, pir-57942, pir-54998) and downregulated piRNAs (e.g., pir-43772, pir-35414, pir-43771) in human follicular fluid, providing a foundation for future research [161].

It is important to note the varying conclusions regarding GC proliferation in PCOS, with some studies reporting inhibited proliferation, while indicate elevated levels. This discrepancy has been attributed to the developmental stage differences of follicles and their contained GCs across studies [109]. It is hypothesized that in PCOS, GCs exhibit a “biphasic alteration”, characterized by increased proliferation and reduced apoptosis in small follicles, and decreased proliferation with increased apoptosis in large follicles [109]. Additionally, certain ncRNAs display complex regulatory effects on GCs, showing contradictory regulatory trends across different studies. For instance, miR-182, a target of circ_0043532, is found at low levels in GCs from patients with PCOS, and its inhibition promotes cell proliferation and cell cycle progression while reducing apoptosis [100]. However, another study reported that miR-182 facilitates GC apoptosis while being downregulated in dihydrotestosterone (DHT)-induced GC PCOS models [162]. circ-FURIN expression is elevated in CCs [105] and the ovarian cortex [106] of patients with PCOS. In one study, circ-FURIN knockdown inhibited proliferation and induced apoptosis in KGN cells [105]. Conversely, in a TTR-induced PCOS model of KGN cells, circ-FURIN knockdown mitigated the TTR-induced proliferation inhibition and apoptosis promotion [106]. These inconsistent findings may be due to whether the cells used in the experiments were in a pathological state. Future research could control for these variables to resolve current controversies.

### Premature Ovarian Failure (POF)

POF, typically characterized by absent menses, hypergonadotropinism, and hypoestrogenism in women under 40 [163], is diagnosed using criteria such as: (i) amenorrhea lasting at least 4 months, (ii) reduced serum E_2_ levels, and (iii) elevated serum FSH levels (exceeding 40 IU/L in at least two samples taken several weeks apart) [164, 165]. The pathogenesis of POF involves a diminished follicle count and/or accelerated follicular depletion during ovarian development [166], with follicular development being closely linked to GCs and regulated by ncRNAs (Fig. 4).

Clinical research has previously identified elevated miR-23a expression in the plasma of patients with POF, with in vitro studies confirming its role in promoting GC apoptosis [166]. Additionally, lncRNA DLEU1 is found to be highly expressed in GCs of patients with POF, contributing to disease progression by enhancing GC apoptosis [88]. High levels of lncRNA FMR6 have also been detected in the follicular fluid and GCs of patients with POF, where it promotes apoptosis and inhibits the proliferation of KGN cells [82].

Animal studies have shown significant downregulation of lncRNA nuclear enriched abundant transcript 1 (NEAT1) in the ovarian tissue of CTX-induced POF mice. In vitro experiments further demonstrated that lncRNA NEAT1 functions as a sponge for miR-654, thereby upregulating STC2 expression, which plays a critical role in inhibiting GC apoptosis and autophagy [123]. Similarly, circ_0002021 is markedly downregulated in the ovarian tissue of CTX-induced POF mice and KGN cells. This circRNA can be delivered to GCs *via* hucMSCs-Exo, where it alleviates GC oxidative stress, regulates the cell cycle, and mitigates cellular senescence [110], thereby improving ovarian function through multiple pathways (Fig. 5).

### Premature Ovarian Insufficiency (POI)

POI is a clinical syndrome marked by the decline of ovarian function before the age of 40, typically presenting with menstrual irregularities (amenorrhea or oligomenorrhea), elevated gonadotropin levels and reduced E_2_ concentrations [167]. Although POI bears similarities to POF, the two conditions, historically used interchangeably, have now been delineated. The diagnosis of POI is primarily based on menstrual disturbances and specific biochemical markers. As per the European Society of Human Reproduction and Embryology (ESHRE) Guideline [167], the diagnostic criteria include: (i) amenorrhea/oligomenorrhea for at least four months, (ii) elevated FSH levels exceeding 25 IU/l on two separate occasions with an interval greater than four weeks.

The pathogenesis of POI, much like that of POF, is associated with a deficiency in primordial follicle pool and/or accelerated follicular depletion [139], which is intricately linked to GC development and ncRNA regulation (Fig. 4). Clinical investigations have demonstrated a reduction in the expression of lncRNA DANCR in the GCs of patients with POI, where its knockdown induces cellular senescence-related phenotypes, including inhibited proliferation, G1 phase arrest, and DNA damage [142]. Animal models further corroborate that lncRNA DANCER knockout leads to decreased fertility, hormonal imbalances, and increased follicular atresia, all characteristic of POI [142]. Additionally, lncRNA PVT1 is markedly downregulated in the ovarian tissue of patients with POI, a condition that facilitates GC apoptosis *via* upregulation of Foxo3a protein levels; conversely, in vivo experiments have demonstrated that lncRNA PVT1 overexpression can restore ovarian function in POI mice [87]. Similarly, linc02690 (lncRNA GCAT1) [114] and lncRNA ZNF674-AS1 [139], both downregulated in the GCs of patients with biochemical POI (bPOI), contribute to the inhibition of GC proliferation and increased follicular atresia. In contrast, lncRNA FMR6, while showing a less pronounced trend of overexpression in GCs of patients with fragile X-associated POI (FXPOI), exhibits a significant negative correlation with the oocyte count [168]. This regulatory mechanism parallels later findings of lncRNA FMR6 overexpression in the follicular fluid and GCs of patients with POF, suggesting its inhibitory role in follicular development and ovarian function [82].

In mouse models of POI induced by lipopolysaccharide (LPS) and cisplatin, both miRNA-146 and lncRNA HOTAIR were observed to be downregulated. These molecules are essential for GC development, with miR-146 enhancing GC vitality and reducing apoptosis by mitigating inflammatory responses [140], and lncRNA HOTAIR promoting GC autophagy and proliferation through regulation of ATG14 protein levels *via* sponging miR-148b-3p [119]. Thus, it can be seen that lncRNA HOTAIR is involved in the regulation of different diseases such as PCOS and POI, demonstrating its multiple regulatory functions (Fig. 5). Furthermore, in POI mice induced by CTX combined with busulfan, miR-122-5p— likely originating from ovarian-derived exosomes— was significantly upregulated, contributing to GC apoptosis by modulating BCL9 [90].

### Diminished Ovarian Reserve (DOR)

DOR refers to a condition in which women of reproductive age exhibit a reduced response to ovarian stimulation or diminished fertility compared to their peers, primarily characterized by a decline in both the quantity and quality of oocytes [169]. DOR often manifests in the early stages of ovarian function decline, and its diagnosis largely depends on abnormal ovarian reserve test results, such as an antral follicle count (AFC) below 5–7 follicles or anti-Müllerian hormone (AMH) levels below 0.5–1.1 ng/ml [165]. Notably, patients with DOR may maintain normal menstrual patterns but experience reduced fertility, with adverse effects on reproductive outcomes [170]. As a result, the pathogenesis of DOR has emerged as a critical focus in the study of early ovarian decline.

Similar to POF and POI, the primary pathological hallmark of DOR is disrupted oocyte and follicle development, which is also influenced to some extent by the developmental status and physiological function of GCs (Fig. 4). Elevated levels of apoptotic proteins have been detected in follicular fluid GCs from patients with DOR, potentially linked to the overexpression of miR-6881-3p and its downregulation of drosophila mothers against decapentaplegic protein (SMAD) 4 in these cells [85]. Furthermore, miR-6881-3p expression has also been positively correlated with basal FSH levels and negatively correlated with ovarian reserve and outcomes of assisted reproductive technology (ART), underscoring its detrimental impact on follicular development [85]. It can be inferred that miR-6881-3p may participate in the pathogenesis of DOR through different pathways such as promoting cell apoptosis and reducing estrogen synthesis (Fig. 5). Similarly, miR-484, which is also overexpressed in GCs of patients with DOR, contributes to ovarian reserve decline, as evidenced by increased FSH levels, decreased AMH, and reduced AFC. It is inversely associated with ART outcomes, such as the number of retrieved oocytes and the proportion of high-quality embryos. In vitro studies suggest that miR-484 may inhibit GC proliferation and induce apoptosis by targeting YAP1 mRNA and causing mitochondrial dysfunction [134]. Additionally, differential expression of miRNAs in follicular fluid-derived exosomes from patients with DOR has been identified, with significant upregulation of miR-28-3p, miR-155-5p, and miR-29a-5p, although their regulatory effects on GCs warrant further investigation [171].

In patients with DOR, certain ncRNAs exhibit a downregulated trend. For instance, miR-106a, which plays a role in enhancing GC vitality and inhibiting apoptosis, is notably underexpressed in both the GC and serum of patients with DOR [86]. This underexpression suggests that the pathogenesis of DOR may involve excessive GC apoptosis. Additionally, lncRNA PWRN1, which is significantly underexpressed in the GCs of patients with DOR, contributes to abnormal follicular development and reduced oocyte quality by inhibiting E_2_ and progesterone synthesis and promoting GC apoptosis. Its detrimental effects on oocyte quantity and embryo implantation have also been confirmed in animal models [112]. Similarly, lnc-GULP1-2:1 is downregulated in luteinized GCs from patients with DOR [115]. Interestingly, in vitro studies reveal that overexpression of lnc-GULP1-2:1 inhibits KGN cell proliferation by affecting the cell cycle [115], suggesting that its downregulation might promote GC proliferation. This finding diverges from other studies where GC proliferation in DOR is typically inhibited by ncRNA, indicating that further research is needed to clarify the role of lnc-GULP1-2:1 in DOR GCs. Transcriptome sequencing combined with RT-qPCR validation has identified several lncRNAs that are significantly downregulated in GCs of patients with DOR and are involved in the regulation of GC proliferation and apoptosis, including lncRNA NEAT1, lncRNA guanine nucleotide-binding protein subunit gamma-12 (GNG12), and lncRNA zinc-finger E-box binding homeobox 2-AS1 (ZEB2-AS1). The biological processes and regulatory targets through which these lncRNAs induce follicular atresia and contribute to DOR could become key areas for future research [172].

Moreover, the differential expression of piRNAs in DOR has attracted attention, with researchers identifying 26 piRNAs that are expressed at significantly different levels between the CCs of patients with DOR and NOR. Among these, 5 piRNAs are downregulated in the DOR group, while the remaining 21 are upregulated [45].

## The Potential Role of ncRNAs in Diagnosis and Treatment of Ovarian Dysfunction

### Biomarkers and Diagnosis

Currently, the diagnosis of ovarian function primarily depends on assessing the patient’s menstrual pattern and ovarian and follicular morphology *via* ultrasound, and serological tests. However, ultrasound findings related to ovarian and follicular morphology are often highly variable, influenced by the operator’s proficiency and the quality of the equipment. Moreover, abnormalities in menstrual patterns and serum steroid hormone levels may only become apparent at later stages of disease progression, limiting their effectiveness in detecting early changes. Additionally, the accuracy of ultrasound and serum steroid tests can be constrained by the specific phase of the menstrual cycle.

Emerging evidence suggests that certain ncRNAs exhibit differential expression in patients with ovarian dysfunction compared to healthy individuals, making them promising biomarkers for disease diagnosis. For example, lncRNA MALAT1, which is overexpressed in GCs of patients with PCOS, shows a positive correlation with reproductive function indicators such as basal estrogen, progesterone, the number of retrieved oocytes, and fertilized ova (2PN) [157]. Furthermore, binary logistic regression analysis indicates that lncRNA MALAT1 expression significantly influences the clinical pregnancy rate in patients with PCOS, and receiver operating characteristic (ROC) curve analysis suggests that lncRNA MALAT1 could serve as a predictor of clinical pregnancy outcomes in these patients, highlighting its potential in diagnosing PCOS and assessing prognosis [157]. Similarly, miR-96-5p is downregulated in the follicular fluid, serum, and GCs in patients with PCOS, as well as in the ovaries of PCOS mice. The ROC curve analysis reveals that miR-96-5p levels in the serum of patients with PCOS differ significantly from those in healthy controls [126]. Additionally, miR-96-5p levels are significantly negatively correlated with serum levels of AMH, prolactin (PRL), TTR, luteinizing hormone (LH), LH/FSH ratio, and TTR/E_2_ ratio, while being positively correlated with serum E_2_ levels [126]. These results suggest that miR-96-5p could emerge as a novel serum biomarker for PCOS. In DOR, the level of miR-6881-3p in human follicular fluid GCs is significantly positively correlated with basal FSH levels and negatively correlated with AMH and AFC; it also shows negative correlations with the number of retrieved oocytes and embryos in IVF/ICSI cycles [85]. This indicates that miR-6881-3p is closely linked to ovarian reserve and may play a diagnostic role in the early detection of DOR, as well as in predicting fertility outcomes in ART. Moreover, lncRNA GCAT1 is significantly downregulated in the GCs of patients with bPOI, with its expression levels showing a significant positive correlation with serum AMH and a significant negative correlation with serum basal FSH [114]. This suggests that lncRNA GCAT1 possesses the characteristics of a biomarker for ovarian reserve, further supporting its potential diagnostic utility.

Additionally, numerous ncRNAs, including those previously mentioned (e.g [82, 109, 112]), exhibit differential expression in patients with ovarian dysfunctions such as PCOS [173], POI [174], POF, and DOR. It is important to note that further research is required for many of these ncRNAs, particularly in terms of understanding their association with established diagnostic indicators, evaluating their area under the ROC curve (AUC), and clarifying essential characteristics such as sensitivity, specificity, positive predictive value, and negative predictive value. Moreover, current research on the differential expression of many ncRNAs in ovarian dysfunction often focuses primarily on ncRNAs within GCs. However, obtaining GCs and measuring ncRNA levels within them in clinical practice can be far more challenging than detecting markers in serum. Therefore, it is crucial to further investigate whether these ncRNAs, which are differentially expressed in GCs, also exhibit differential expression in more accessible samples, such as serum. Given that these ovarian dysfunctions significantly impact fertility and reproductive outcomes—areas of great concern to both patients and gynecologists—it is also essential to explore the potential of ncRNAs as biomarkers in predicting fertility outcomes. This would provide a more comprehensive assessment of their diagnostic and clinical application value as new indicators.

### Therapeutic Applications

Currently, the primary treatment for ovarian dysfunctions involves sex hormone therapy, and when fertility issues are present, ART is often required. However, fertility restoration remains a challenge, frequently resulting in suboptimal reproductive outcomes despite ART intervention. Some ncRNAs that have been identified as differentially expressed in patients have shown potential in correcting pathological conditions and improving ovarian function. Human mesenchymal stem cells (hMSCs) offer promising new avenues for treating POF due to the various ncRNAs they carry. For example, miR-126-3p-hucMSCs-exosomes have been shown to increase E_2_ and AMH levels, reduce FSH levels, enhance body and reproductive organ weight, and increase the follicle count in POF model rats [104]. Additionally, hucMSCs-exosomes containing circ_0002021 can significantly alleviate ovarian senescence, correct serological abnormalities (such as inducing an AMH and E_2_ rebound and a decline in FSH), and restore ovarian structure and function in POF mice [110]. Similarly, lncRNA PVT1, when overexpressed *via* vector injection, has been found to repair ovarian function, reduce the number of atretic follicles, elevate E_2_ levels, and decrease FSH levels in POI mice [87]. Furthermore, miR-21 carried by hucMSCs-exosomes has demonstrated the ability to promote GC estrogen secretion in vitro, indicating the therapeutic potential for improving ovarian function [127].

In the context of PCOS treatment, the efficacy of certain ncRNAs has also been explored. linc00173, which is highly expressed in the GCs of patients with PCOS, was effectively targeted in PCOS rats using tail vein injection of sh-linc00173, correcting abnormal serum hormone levels associated with PCOS, including significantly elevated TTR, LH, and E_2_ levels, along with significantly reduced FSH levels [99]. Similarly, si-HOTAIR has been shown to reduce serum TTR, E_2_, and LH levels while increasing FSH levels in PCOS rats, as well as promoting the recovery of ovarian morphology and the estrous cycle, thereby significantly improving the pathological manifestations of PCOS [120]. Overexpression of miR-96-5p has also been found to reverse PCOS-like phenotypes induced by DHEA in mice, including the arrest of the estrous cycle, abnormal ovarian morphological changes, reduced ovarian corpus luteum, increased antral follicles, and elevated TTR levels [126].

Several other ncRNAs have demonstrated protective or therapeutic effects on GC function under disease conditions in vitro or in animal experiments. For instance, linc00092 in extracellular vesicles derived from follicular fluid attenuated DHEA-induced apoptosis in GCs (PCOS models) [92], while the downregulation of circ_FURIN alleviated proliferation inhibition and apoptotic hyperactivity in TTR-induced GCs (PCOS models) [106]. Additionally, upregulation of miR-146 increased viability and attenuated inflammatory responses in LPS-induced GCs (POI models) [140]. However, due to the lack of observations related to sex hormones and other indicators that visually reflect reproductive endocrine function, it remains challenging to establish the direct restorative impact of these ncRNAs on ovarian function and their therapeutic efficacy in treating diseases. Future research should be more comprehensive, incorporating observations on steroid synthesis, the proportion of atretic follicles, changes in ovarian structure, and other relevant indicators to provide robust evidence for the therapeutic potential of these ncRNAs.

## Conclusions

ncRNAs play a critical role in regulating various biological processes in the human body. Specifically, ncRNAs associated with GCs are involved in essential cellular activities such as proliferation, apoptosis, autophagy, cell cycle, steroidogenesis, and so on. Through their regulatory effects on GCs, these ncRNAs contribute to the pathogenesis of ovarian dysfunctions, including PCOS, POF, POI, and DOR. Their potential diagnostic and therapeutic value, however, still requires extensive research.

Recent advancements in molecular biology techniques and the diversification of research methods have enabled a deeper understanding of the roles of ncRNAs in ovarian dysfunction, revealing the complexity and interconnectedness of their disease-regulating effects (Fig. 5). Some ncRNAs have been shown to significantly impact ovarian dysfunction, as evidenced by their differential expression between patients and healthy controls, their regulatory effects on ovaries and GC structure and function, and their efficacy in mitigating disease symptoms in both in vitro and in vivo experiments. Research designs that integrate clinical trials with laboratory experiments may accelerate the translation of basic research findings into clinical applications. In terms of research methodology, many studies utilize bioinformatics analysis to predict ncRNA targets and employ techniques such as dual-luciferase reporter assays, RNA pull-down, and RNA immunoprecipitation to identify binding sites between ncRNAs and their targets. These interactions are then confirmed through in vitro experiments and rescue experiments, which further elucidate pathways and mechanisms by which ncRNAs exert their effects. Some studies systematically explore and demonstrate the regulatory effects of ncRNAs by combining various methods, thereby revealing their influence on multiple GC phenotypes. This comprehensive approach helps to better understand the role of ncRNAs in the development of ovarian dysfunctions. Moreover, emerging research is beginning to shed light on the role of piRNAs in the female reproductive system, with some studies already exploring their differential expression in patients with PCOS or DOR.

## Future Perspectives

Currently, many studies using GCs derived from patients or model animals often overlook the impact of the follicular development stage on GC physiological characteristics, potentially contributing to inconsistent research outcomes—discrepancies already noted in some publications, which require further clarification through additional research. Additionally, conflicting conclusions have emerged in studies examining the same ncRNA. Variability in experimental conditions, such as whether GCs are induced into pathological states through drug treatments or remain untreated, suggests that the physiological or pathological state of the experimental material could significantly influence research results. This factor, along with its potential to induce limitations or errors, necessitates careful consideration in future investigations. Regarding disease research, PCOS is relatively well-documented, whereas the role of ncRNAs in diseases associated with ovarian function decline demands more focused attention. Research into ncRNAs as diagnostic biomarkers remains in its early stages, with limited studies evaluating the accuracy of ncRNAs as diagnostic indicators through rigorous diagnostic tests. The therapeutic application of ncRNAs for ovarian dysfunction is also predominantly in the preclinical phase. However, certain ncRNAs that have been extensively studied both in vivo and in vitro may warrant clinical research evaluation in the future. This review also highlights several ncRNAs, particularly piRNAs, that are still in the early stages of research. At present, their differential expression in patients is recognized, but more in-depth exploration of their regulatory effects on GCs and their involvement in the disease pathogenesis is needed. Such research would provide a stronger foundation for understanding their biological functions and the mechanisms underlying ovarian dysfunctions.

## Abbreviations

AFC: Antral follicle count
AMH: Anti-Müllerian hormone
ART: Assisted reproductive technology
CC: Cumulus granulosa cell
ceRNA: Competitive endogenous RNA
circRNA: Circular RNA
CTX: Cyclophosphamide
DHEA: Dehydroepiandrosterone
DHT: Dihydrotestosterone
DOR: Diminished ovarian reserve
E_2_: estradiol
FSH: Follicle-stimulating hormone
GC: Granulosa cell
hMSCs: Human mesenchymal stem cells
hucMSCs: Human umbilical cord mesenchymal stem cells
LH: Luteinizing hormone
lncRNA: Long non-coding RNA
miRNA: microRNA
ncRNA: Non-coding RNA
PCOS: Polycystic ovary syndrome
piRNA: Piwi-interacting RNA
POF: Premature ovarian failure
POI: Premature ovarian insufficiency
PRL: Prolactin
shRNA: Small hairpin RNA
siRNA: Small interfering RNA
sncRNA: Small non-coding RNA
TTR: Testosterone
